# Exported J domain proteins of the human malaria parasite

**DOI:** 10.3389/fmolb.2022.978663

**Published:** 2022-08-31

**Authors:** Shaikha Y. Almaazmi, Harpreet Singh, Tanima Dutta, Gregory L. Blatch

**Affiliations:** ^1^ Biomedical Research and Drug Discovery Research Group, Faculty of Health Sciences, Higher Colleges of Technology, Sharjah, United Arab Emirates; ^2^ Department of Bioinformatics, Hans Raj Mahila Maha Vidyalaya, Jalandhar, India; ^3^ Vice Chancellery, The University of Notre Dame Australia, Fremantle, WA, Australia; ^4^ The Institute of Immunology and Infectious Diseases, Murdoch University, Perth, WA, Australia; ^5^ PathWest Nedlands, QEII Medical Centre, Nedlands, WA, Australia; ^6^ Biomedical Biotechnology Research Unit, Department of Biochemistry and Microbiology, Rhodes University, Grahamstown, South Africa

**Keywords:** heat shock proteins, J domain proteins, molecular chaperones and co-chaperones, *Plasmodium falciparum*, protein export, protein folding

## Abstract

The heat shock protein 40 (Hsp40) family, also called J domain proteins (JDPs), regulate their Hsp70 partners by ensuring that they are engaging the right substrate at the right time and in the right location within the cell. A number of JDPs can serve as co-chaperone for a particular Hsp70, and so one generally finds many more JDPs than Hsp70s in the cell. In humans there are 13 Hsp70s and 49 JDPs. The human malaria parasite, *Plasmodium falciparum*, has dedicated an unusually large proportion of its genome to molecular chaperones, with a disproportionately high number of JDPs (PfJDPs) of 49 members. Interestingly, just under half of the PfJDPs are exported into the host cell during the asexual stage of the life cycle, when the malaria parasite invades mature red blood cells. Recent evidence suggests that these PfJDPs may be functionalizing both host and parasite Hsp70s within the infected red blood cell, and thereby driving the renovation of the host cell towards pathological ends. PfJDPs have been found to localize to the host cytosol, mobile structures within the host cytosol (so called “J Dots”), the host plasma membrane, and specialized structures associated with malaria pathology such as the knobs. A number of these exported PfJDPs are essential, and there is growing experimental evidence that they are important for the survival and pathogenesis of the malaria parasite. This review critiques our understanding of the important role these exported PfJDPs play at the host-parasite interface.

## Introduction

**FIGURE 1:**
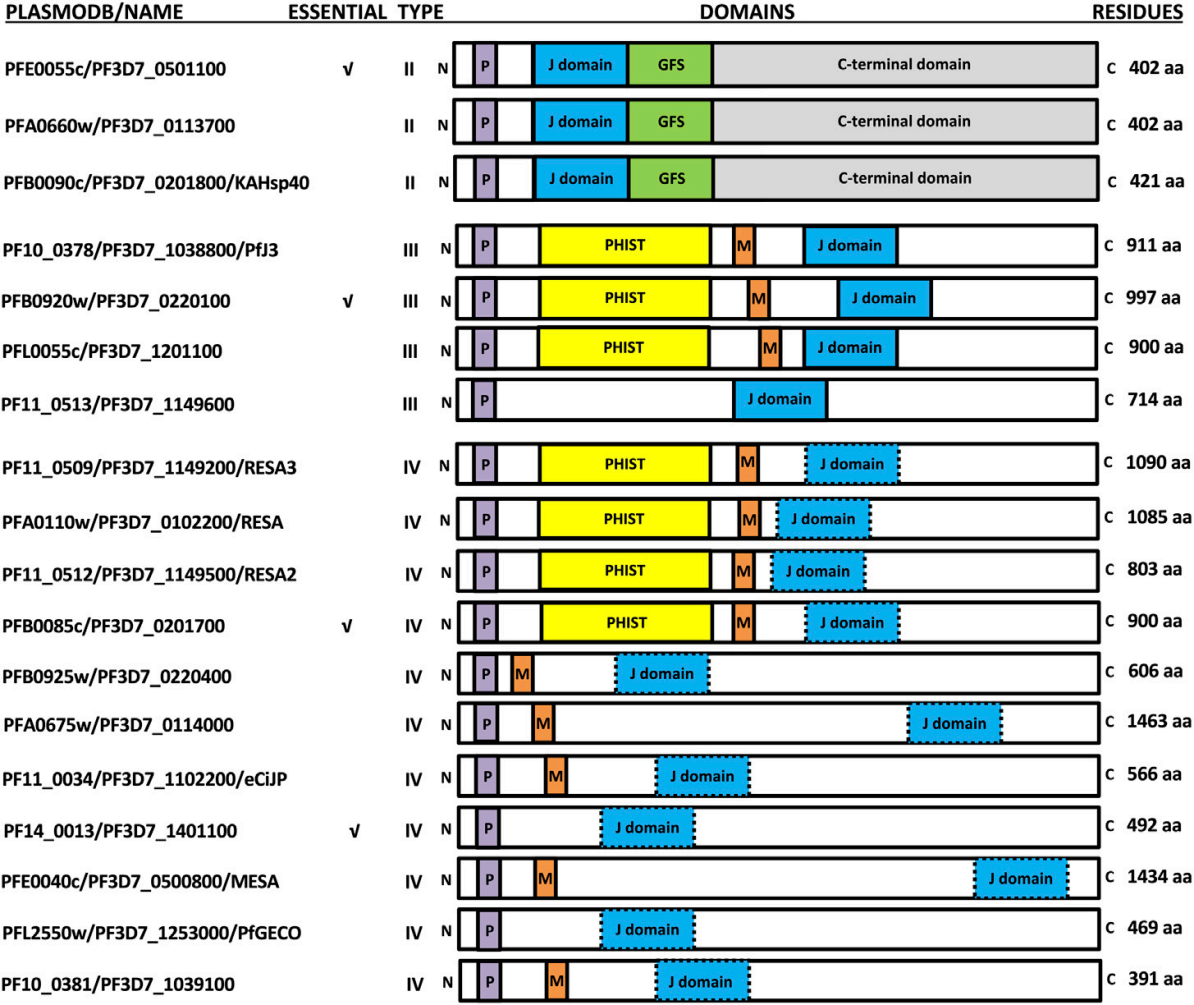
Schematic representation of the exported PfJDPs. The PlasmoDB accession numbers/common name, essential nature, type, domain organization (not to scale), and number of amino acids are shown. The domains of the PfJDPs shown are the signature J domain (blue) which stimulates the ATPase activity of Hsp70, the glycine/phenylalanine/serine (GFS)-rich region (green) involved in regulation of protein substrate binding by Hsp70, the C-terminal domain (grey) involved in substrate binding and dimerization, the PEXEL motif required for export through the PTEX, and the PHIST domain (yellow) and MEC motif (red) involved in binding to the erythrocyte cytoskeleton. Certain PfJDPs (type IVs) have a J domain where the highly conserved histidine-proline-aspartic acid (HPD) motif is altered (blue with dashed border).

The malaria parasite, *Plasmodium falciparum*, invades the cells of its human host, enabling it to evade the immune system and ultimately harness the cellular machinery to propagate itself and cause severe pathology. Surrounded by a self-created parasitophorous vacuole (PV) within human erythrocytes, the malaria parasite renovates the host cell by exporting over 400 parasite proteins, including a number of heat shock proteins, which oversee protein folding as molecular chaperones and co-chaperones (Hiller et al., 2004; Marti et al., 2004; Jonsdottir et al., 2021). An important co-chaperone family, the *P. falciparum* J domain proteins (PfJDPs; also called heat shock protein 40 s, Hsp40s), are localized to almost every compartment of the infected erythrocyte, with arguably the greatest number of exported members of any protein family (Sargeant et al., 2006; Botha et al., 2007; Dutta et al., 2021a; Figure 1). Furthermore, many of the exported PfJDPs are expressed in the early stages of the asexual phase of the malaria parasite life cycle (Bozdech et al., 2003), consistent with their proposed critical role in the export of other malaria proteins and survival during febrile episodes (Dutta et al., 2021a). Indeed, there is growing evidence that these exported PfJDPs are key players in the survival and pathogenesis of the malaria parasite. The following sections of this review provide a critique of our understanding of the important role that these exported PfJDPs play at the host-parasite interface of malaria pathology.

## The diversity, structure and function of PfJDPs

There are at least 49 PfJDPs encoded on the *P. falciparum* genome, far more than any other *Plasmodium* species (Njunge et al., 2013). All JDPs by definition contain a signature J domain (Kampinga et al., 2019), which contains a highly conserved histidine-proline-aspartic acid (HPD) motif, and is essential for regulation of the chaperone activity of partner heat shock protein 70 s (Hsp70s) (Hennessy et al., 2005). There are a number of other domains which have been used to categorize JDPs into four types (types I-IV; Botha et al., 2007). Interestingly, the HPD motif was thought to be invariant, until the discovery of the type IV JDPs containing a non-conserved HPD motif (Botha et al., 2007).

The *Plasmodium* export element (PEXEL; Hiller et al., 2004; Marti et al., 2004) has been shown to tag many *P. falciparum* proteins for export through the *Plasmodium* translocon of exported proteins (PTEX; de Koning-Ward et al., 2009; Beck et al., 2014; Elsworth et al., 2014; Elsworth et al., 2016). While the PEXEL was initially identified on 19 PfJDPs (Botha et al., 2007; Njunge et al., 2013; Pesce and Blatch 2014), recent revisions have indicated 18 PEXEL-containing PfJDPs (Dutta et al., 2021a). Therefore, at least 18 PfJDPs are proposed to be exported into the infected erythrocyte based on the presence of a PEXEL; three type II, four type III and eleven type IV PfJDPs (Figure 1).

The exported PfJDPs appear to be critical for survival and pathogenesis of the malaria parasite, through their essential nature (4/18; Zhang et al., 2018; Figure 1), involvement in protein folding of exported virulence proteins (e.g., PFE0055c and PFA0660w associated with “J Dots”; Külzer et al., 2010; Külzer et al., 2012; Behl et al., 2019), requirement for growth or survival under febrile conditions (e.g., PFA0110w, the ring-infected erythrocyte surface antigen protein, RESA; Silva et al., 2005; Diez-Silva et al., 2012) and involvement in pathogenesis (e.g., PF10_0381; knockout causes loss of knobs; Maier et al., 2008). Of the 6 PfHsp70s expressed by the malaria parasite (Shonhai et al., 2007; Przyborski et al., 2015; Shonhai et al., 2021), PfHsp70-x appears to be the only member exported into the cytosol of the infected host erythrocyte (Külzer et al., 2010; Külzer et al., 2012; Grover et al., 2013), even though it lacks a PEXEL motif (Rhiel et al., 2016). Interestingly, the host cytosol appears to contain human chaperones and co-chaperones, including JDPs (which are likely to be non-functional remnants; Pasini et al., 2006; van Gestel et al., 2010) and significant levels of functional human Hsp70. (e.g., HSPA1A, also called Hsp72; referred to here as hHsp70), occurring free or in complex with Hsp90 and the Hsp70/Hsp90 organising protein, Hop (Banumathy et al., 2002). Furthermore, evidence is emerging that certain exported PfJDPs are capable of functionally interacting with PfHsp70-x, or hHsp70, or both Hsp70s (Dutta et al., 2021a; Diehl et al., 2021).

**FIGURE 2:**
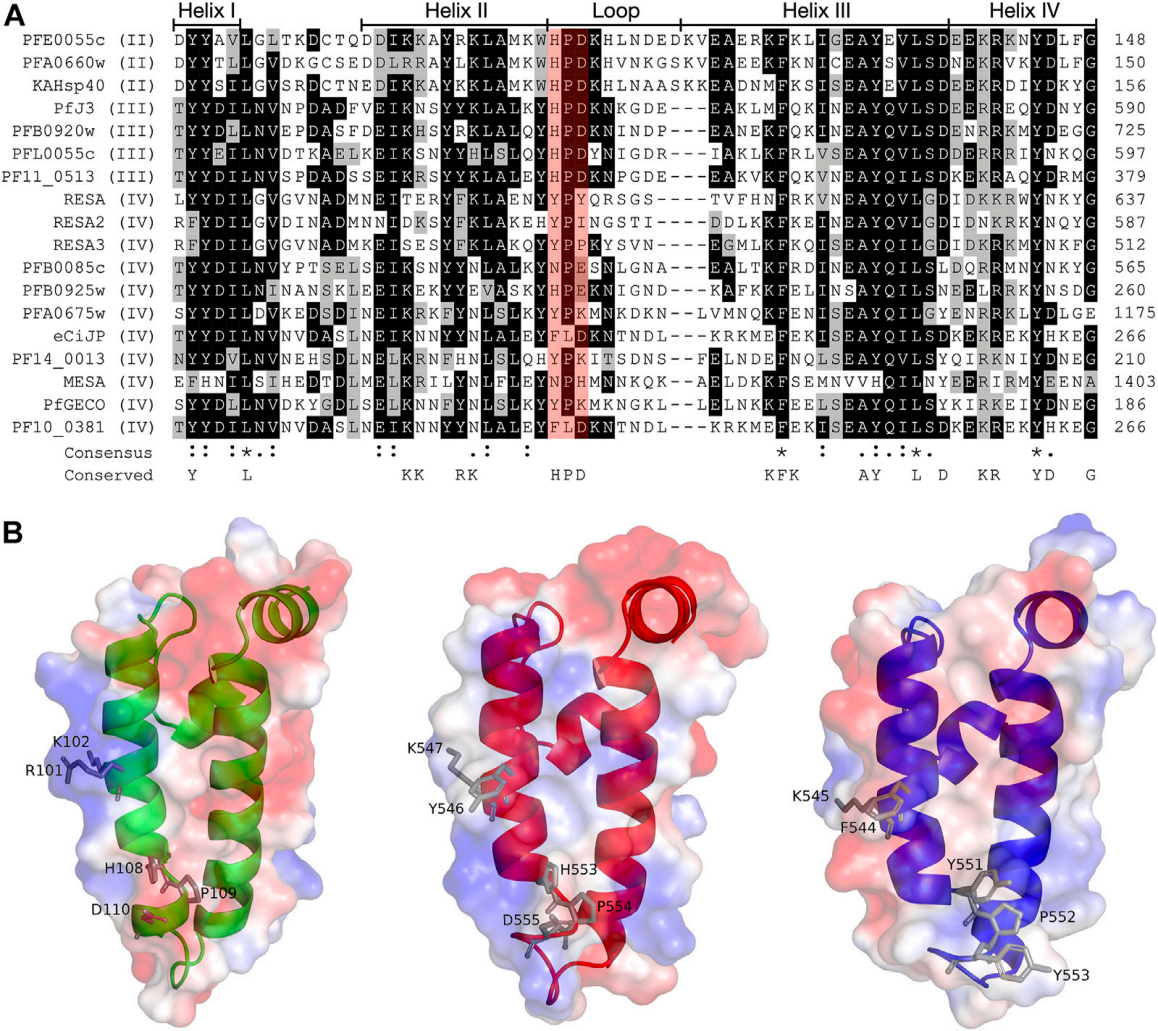
Multiple sequence alignment and molecular modelling of the J domains of exported PfJDPs. **(A)** Multiple sequence alignment of the J domains of the 18 exported PfJDPs proteins. The proteins are defined by either their PlasmoDB accession number or common name in the first column, and the roman numerals in brackets refer to the type of JDP. Colored in black are identical amino acids (in at least 50% of the aligned sequences), colored in light grey are similar amino acids (in at least 50% of the aligned sequences), and colored in white are the amino acids with no identity or similarity. The default categories for similar amino acids were applied to the multiple sequence alignment (ILV, FWY, KRH, DE, GAS, P, C and TNQM). The row titled “Consensus” are the common consensus symbols of the multiple sequence alignment: an * (asterisk) indicates positions which have a single, fully conserved residue; a: (colon) indicates conservation between groups of strongly similar properties; and a. (period) indicates conservation between groups of weakly similar properties. The row titled “Conserved” refers to residues previously found to be highly conserved across J domains of different origins, with the residues defined at the bottom of the alignment (Hennessy et al., 2000; Hennessy et al., 2005). The protein helices and loop region are defined by bidirectional lines on top of the alignment. Highlighted in red shading is the HPD motif. The alignment was created using Clustal Omega (Sievers and Higgins, 2018) and rendered with box shading using Multiple Align Show (Stothard, 2000). **(B)** Three dimensional models of the J domains of PFE0055c type II (green), PfJ3 type III (red), and RESA type IV (blue) to illustrate the conserved HPD and RK motif (grey sticks). The positive charge is shown in blue colored surface, the negative charge is shown in red colored surface and the neutral potential are shown in white colored surface. The surface electrostatic potential was calculated by APBS. The models were prepared using SWISS-MODEL (Waterhouse et al., 2018; the template structures are listed in Supplementary Table S1) and graphically rendered using PyMol 2.5.2 (PyMOL Molecular Graphics System, Version 2.0 Schrödinger, LLC).

The structures of the ATPase (Day et al., 2019) and substrate binding (Schmidt and Vakonakis, 2020) domains of PfHsp70-x have been elucidated, as has the structure of the J domain of the exported PfJDP, PFA0660w (Day et al., 2019). In addition, molecular modelling revealed that helix II of the PFA0660w J domain makes a primary interface with the ATPase domain of PfHsp70-x (Day et al., 2019). Comparative molecular modelling suggested that the PFA0660w J domain-PfHsp70-x complex was less stable than that of another exported PfJDP (PFE0055c J domain-PfHsp70-x complex) (Dutta et al., 2021b). The functional differences between PFA0660w and PFE0055c were attributed to the J domain helix II of PFA0660w being less positively charged than the more typical J domain of PFE0055c. Interestingly, a multiple sequence alignment and surface electrostatic potential analysis of the J domains of all the exported PfJDPs revealed that the positive nature of the J domain helix II appears to decrease going from type II to type IV, with the type IVs exhibiting significant negative charge or hydrophobicity (Figure 2; Supplementary Figure S1). These J domain surface differences together with the considerable variations in the HPD motifs for the type IVs, suggests that the structural and functional nature of the association, if any, between the different types of exported PfJDPs and Hsp70 (PfHsp70-x or hHsp70) are likely to be considerably different.

## J Dots in the host cytosol contain PfJDPs and PfHsp70-x

J Dots are highly mobile lipid-containing protein complexes found within the malaria parasite-infected erythrocyte cytosol (Külzer et al., 2010; Külzer et al., 2012; Petersen et al., 2016). Localization and immunoprecipitation studies have convincingly detected the exported type II PfJDPs, PFA0660w and PFE0055c, and PfHsp70-x in J Dots, which suggested that they formed a functional partnership in the host cytosol. However, these may not be the only J Dot chaperone partnerships, as an exported type IV PfJDP, called eCiJP (PF11_0034; a paralogue of PF10_0381), has recently been reported to localize to J-Dots (Sahu et al., 2022). There is also evidence that J Dots associate with *P. falciparum* erythrocyte membrane protein 1 (PfEMP1), leading to the proposal that they may be involved in the trafficking of this major virulence factor (Külzer et al., 2012). These findings have been corroborated by biochemical studies on PFA0660w, PFE0055c and PfHsp70-x. PFA0660w was found to be highly effective at protein aggregation suppression, and both PFA0660w and PFE0055c were shown to be capable of specifically stimulating the basal ATPase activity of PfHsp70-x and not hHsp70 (Daniyan et al., 2016; Daniyan and Blatch 2017; Dutta et al., 2021b). These steady-state ATPase assays were conducted under saturating ATP concentrations which favour chaperone-co-chaperone over chaperone-substrate interactions, and in the case of PFE0055c could be inhibited by a J domain-specific inhibitor (chalcone C86), suggesting that PFA0660w and PFE0055c form specific co-chaperones partnerships with PfHsp70-x. PFE0055c stimulated the ATPase activity to a much greater extent than PFA0660w, and molecular modelling suggested that PFE0055c formed a more stable complex with PfHsp70-x than did PFA0660w (Dutta et al., 2021b). Furthermore, biochemical studies have shown that PfHsp70-x was capable of simultaneously associating with cholesterol-bound PFA0660w and PfEMP1 to form a stable complex (Behl et al., 2019). In contrast, biochemical studies using just the J domain of PFA0660w and PFE0055c (Day et al., 2019) or a PFA0660w J domain-substrate fusion construct (Diehl et al., 2021) have provided evidence that they may be capable of functional interaction with both PfHsp70-x and hHsp70. The inconsistency between the biochemical studies on full-length versus J domain proteins with respect to the hHsp70 findings, could be due to differences in the nature of the assays. However, it is also likely that the full-length PfJDPs contain regions beyond the J domain that determine co-chaperone-chaperone specificity. This interpretation is consistent with structural analyses of the interaction between PFA0660w and PfHsp70-x, which indicated that they associated in a bipartite manner requiring both the J domain and the G/F region of PFA0660w (Behl and Mishra 2019).

## Exported PfJDPs play a key role in malaria pathology

The third exported type II PfJDP (PFB0090c, also called knob-associated Hsp40, KAHsp40; Acharya et al., 2012), has not been reported to associate with PfHsp70-x or with J Dots, despite significant structural similarity to the other type II PfJDPs. In contrast, PFB0090c has been shown to associate with PTEX and knobs, and is proposed to be involved in the trafficking, folding, and assembly of knob protein complexes (Acharya et al., 2012). Knob complexes appear to contain human chaperones and co-chaperones in a highly complexed state (Hsp70, Hsp90 and Hop), and there is evidence that hHsp70 plays a role in assembly of knobs (Banumathy et al., 2002; Alampalli et al., 2018). It is tempting to speculate that PFB0090c is the co-chaperone recruiting hHsp70 to knobs. However, knock-out studies and associated genetic and biochemical analyses suggested that PFA0660w was also involved in knob formation and cytoadherence in collaboration with hHsp70 (Diehl et al., 2021). Interestingly, all of the exported type II PfJDPs (PFB0090c, PFA0660w, and PFE0055c) have been shown to functionally interact with hHsp70 using a heterologous yeast two-hybrid complementation assay (Jha et al., 2017). However, this assay did not test for functional association between PfJDPs and PfHsp70-x, and the biochemical and biophysical nature of the interactions were not investigated.

There are four exported type III PfJDPs (PFB0920w, PFL0055c, PF10_0378/Pfj3 and PF11_0513), three of which contain the *Plasmodium* helical interspersed sub-telomeric (PHIST) domain (Sargeant et al., 2006; Oakley et al., 2007; Oakley et al., 2011; Figure 1). Four exported type IV PfJDPs (PF11_0509/RESA3, PF11_0512/RESA2, PFA0110w/RESA and PFB0085c; Figure 1) also contain the PHIST domain (Figure 1). All of these PHIST-containing PfJDPs also contain the MEC (MESA erythrocyte cytoskeleton-binding) motif found in five other exported type IV PfJDPs (PFE0040c/mature parasite-infected erythrocyte surface antigen [MESA], PFA0675w, PFB0925w, PF10_0381, PF11_0034/eCiJP; Bennett et al., 1997; Kilili and LaCount 2011; Njunge et al., 2013; Figure 1; Supplementary Figure S2). PHIST proteins are proposed to play an important role in trafficking of PfEMP1 proteins and the modulation of membrane rigidity and cytoadherence in parasite-infected erythrocytes (Kumar et al., 2019). Therefore, the presence of the MEC motif and the PHIST domain in these PfJDPs, is consistent with the proposed role of many of these proteins in remodelling at the cytoskeleton-membrane interface of the infected erythrocyte. RESA is arguably the most extensively studied exported PfJDP, and has been shown to play a key role in modulation of the rigidity of the infected erythrocyte membrane (Silva et al., 2005; Maier et al., 2008). RESA associates with spectrin and stabilizes its tertiary structure (Pei et al., 2007), and its effect on the cytoskeleton decreases the deformability of the infected erythrocyte, and protects the membrane during febrile episodes (Silva et al., 2005; Mills et al., 2007; Diez-Silva et al., 2012). The MEC motif enables MESA to bind to erythrocyte protein 4.1, thereby tethering it to the cytoskeleton (Coppel 1992; Bennett et al., 1997; Waller et al., 2003; Black et al., 2008); and MESA has been implicated in erythrocyte membrane modification (Coppel et al., 1988). However, the absence of MESA does not seem to have an influence on cytoadhesion (Petersen et al., 1989; Cooke et al., 2002). PF10_0381 has been implicated in knob formation (Maier et al., 2008), while its paralogue, eCiJP, has been shown to associate with the erythrocyte cytoskeleton, and potentially recruit hHsp70 to this location (Sahu et al., 2022; Figure 1). Apart from this report for eCiJP, there are virtually no reports on the interaction of type IV PfJDPs with Hsp70s (PfHsp70-x or hHsp70). Whether these proteins function as co-chaperones of Hsp70s remains to be determined.

## Exported PfJDPs as drug targets

**FIGURE 3:**
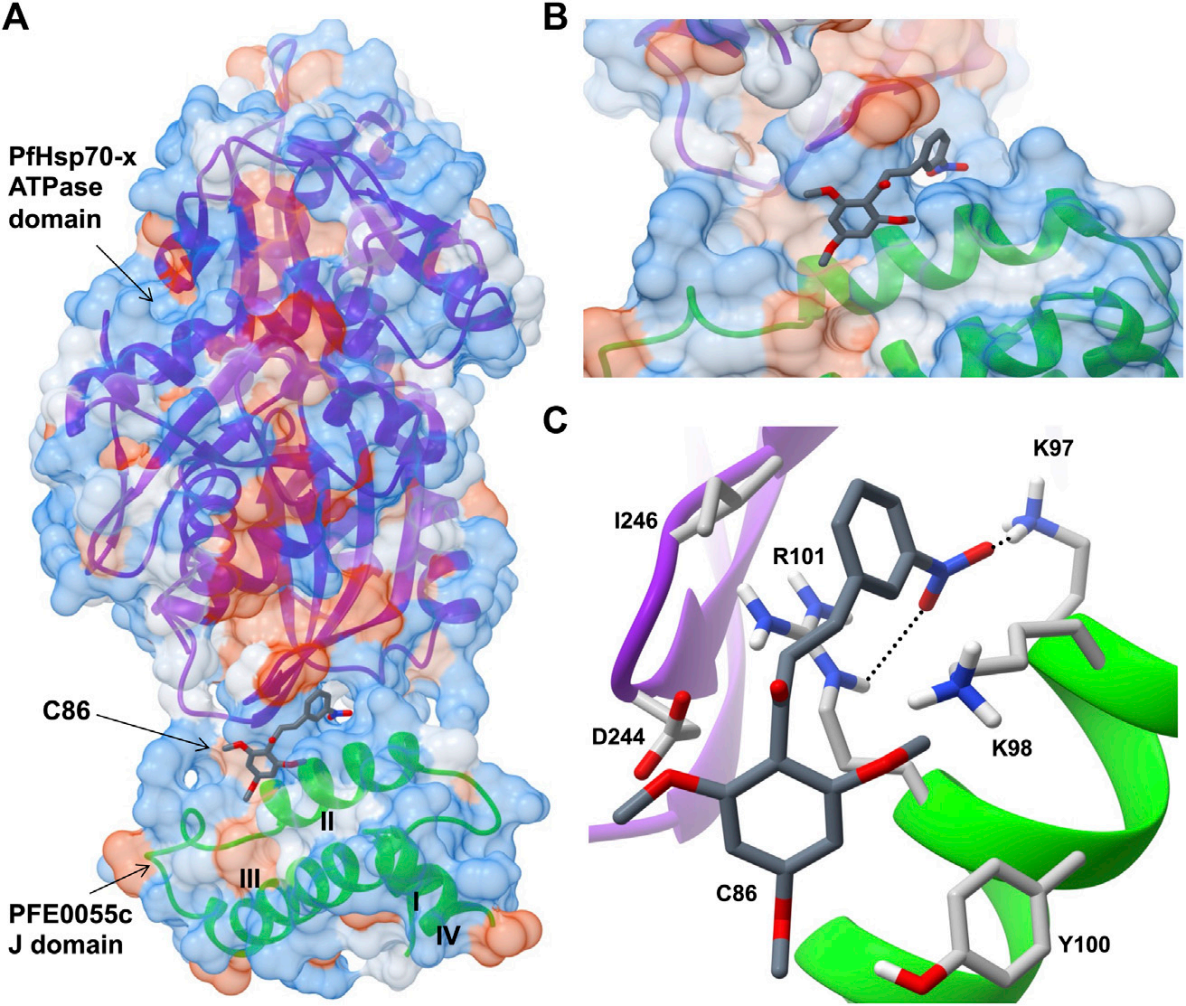
Predicted model of a complex of the small molecule inhibitor C86, PFE0055c J domain and PfHsp70-x ATPase domain. **(A)** Overall binding pose of C86 (middle) to the complex formed between the PfHsp70-x ATPase domain (top) and the PFE0055c J domain (bottom). **(B)** Focused view of c86 binding to the complex. **(C)** Detailed view of residues interacting with C86. The surface (set at 60% transparency) is colored according to amino acid hydrophobicity using the Kyte-Doolittle scale (with dodger blue representing more hydrophilic, to white, to orange red representing more hydrophobic residues). The cartoon representation underneath the surface depicts the secondary structure elements in the PfHsp70-x ATPase domain (purple) and PFE0055c J domain (green). The roman numerals (I to IV) mark the four helices of the PFE0055c J domain. C86 as well the interacting residues of the complex have been shown using the element color scheme (using red, blue and grey for oxygen, nitrogen and carbon, respectively). The carbons in C86 are depicted using dark grey while its interacting residues are colored in light grey. The residues are shown as sticks, and the numbering refers to their position in the full length protein. The predicted hydrogen bonds were identified using LigPlot + version 2.2.5 (https://www.ebi.ac.uk/thornton-srv/software/LigPlus/; Laskowski and Swindells, 2011), and have been rendered using FindHBond option in Chimera in relaxed mode and depicted using dotted lines (black). The lengths of the larger and the smaller hydrogen bonds are 2.74 Å and 2.32 Å, respectively. The PfHsp70-x ATPase domain-PFE0055c J domain complex was obtained using HADDOCK2.2 (Dutta et al., 2020a) while AutoDock Vina (https://vina.scripps.edu/; Trott and Olson, 2010) was used to dock C86 into this complex. Images for the 3D structures were rendered using UCSF Chimera 1.10.1 (https://www.cgl.ucsf.edu/chimera/), using one of the nine best binding conformations of C86.

Very few small molecule inhibitors have been identified that bind specifically to JDPs (e.g., phenoxy-N-arylacetamides; Cassel et al., 2012). In addition, the identification of inhibitors that can directly disrupt the interaction between JDPs and Hsp70s is challenging, as the interaction is transient and the binding sites are located on the surface of the protein as shallow exposed clefts which are recalcitrant to small molecule association (Pesce et al., 2016). Nevertheless, recent advances in our understanding of PfJDP-PfHsp70 interactions and their inhibition, suggests that they are a promising target for anti-malarial drug development (Daniyan and Blatch, 2017; Dutta et al., 2021a). Pyrimidinones have shown potential as protein-protein interaction inhibitors when tested on the parasite-resident PfJDP-PfHsp70 system (Botha et al., 2011). In contrast, naphthoquinones and prenylated alkaloids were identified that functionally disrupted the exported PfHsp70-x and its interaction with JDPs (Cockburn et al., 2014). The most promising inhibitor was the prenylated alkaloid, malonganenone A (MalA), where side-by-side inhibition assays conducted on JDP-PfHsp70-x and JDP-hHsp70 showed that MalA had no effect on the basal ATPase activity of both Hsp70s, and yet significantly inhibited the JDP-stimulated ATPase activity of PfHsp70-x but not that of hHsp70 (Cockburn et al., 2014). MalA has significant anti-malarial activity, but low cytotoxicity to human cell lines (Cockburn et al., 2011), suggesting that it is a promising small-molecule candidate for the development of specific inhibitors of the exported PfJDP-PfHsp70-x system. Chalcones represent another class of small molecules found to have anti-malarial activity (Sinha et al., 2019; Qin et al., 2020), and to inhibit JDP-hHsp70 interaction (Moses et al., 2018). Interestingly, chalcone C86 (3-nitro-2′,4′,6′-trimethoxychalcone) was found to bind to the J domain of JDP families A-C, indicating that it was a pan-inhibitor of JDPs (Moses et al., 2018). Recently, it was found that C86 also inhibited PfJDP-PfHsp70-x interaction (Dutta et al., 2021b). While C86 had no effect on the basal ATPase activity of PfHsp70-x, it significantly inhibited the PFE0055c-stimulated ATPase activity of PfHsp70-x (Dutta et al., 2021b). Notably, significant inhibition was only observed when C86 was pre-incubated with PFE0055c prior to the addition of PfHsp70-x. This finding is consistent with C86 binding specifically to the region of the J domain of PFE0055c that makes contact with PfHsp70-x (Dutta et al., 2021a). Molecular modelling of a predicted complex suggests that C86 makes major contacts with previously identified residues of helix II of the PFE0055c J domain involved in binding to the PfHsp70-x ATPase domain (Figure 3). This model provides a rationale for the development of C86 chemotypes, with the aim of identifying high affinity derivatives with specificity for PfJDPs. Given that the exported PfJDP, PFE0055c, is essential (Zhang et al., 2018), the inhibition of its functional interaction with PfHsp70-x represents an important interface for anti-malarial drug development.

## Conclusion

There is solid evidence that certain exported type II PfJDPs (PFA0660w and PFE0055c) functionally associate with PfHsp70-x (Daniyan et al., 2016; Dutta et al., 2021b), and are core components of J Dots potentially involved in the trafficking and folding of virulence factors (Külzer et al., 2010; Külzer et al., 2012; Petersen et al., 2016; Behl et al., 2019). However, there is also emerging evidence that these PfJDPs play a role in knob formation (PFB0090c, Acharya et al., 2012), potentially in association with hHsp70 (PFA0660w, Diehl et al., 2021). Furthermore, a type IV PfJDP (eCiJP) was recently reported to associate with both J Dots and the erythrocyte cytoskeleton, with the latter potentially involving interaction with hHsp70 (Sahu et al., 2022). Based on the presence of the PHIST domain and MEC motif, it would not be surprising if many of the exported type III and type IV PfJDPs are found to associate at the cytoskeleton-membrane interface, and to be involved in recruitment of hHsp70 for remodeling purposes. Detailed mechanistic studies will no doubt shed light on this fascinating host-parasite interface, and elucidate the novel manner in which these exported PfJDPs interact with their partner Hsp70s (PfHsp70-x or hHsp70) to trigger the assembly of erythrocyte cytoskeleton and knob-associated proteins. While there is a need to continue exploring the role of exported PfJDPs in *P. falciparum*-infected erythrocytes, there is a pressing need to investigate their role in other stages of the parasite life cycle. For example, apart from studies on the type IV PfJDP, *P. falciparum* gametocyte erythrocyte cytosolic protein (PfGECO/PFL2550w; Morahan et al., 2011; Figure 1), there have been limited studies on PfJDPs expressed in the gametocyte stage. Given the role of these exported PfJDPs in the pathogenesis of malaria, and the evidence indicating that their function can be inhibited, the elucidation of their protein networks across the entire parasite life cycle is needed to fully understand their biological role, and to reveal further novel anti-malarial drug targets.
