# Supplementary material for: Exported J domain proteins of the human malaria parasite
Source: Front Mol Biosci. 2022 Aug 31;9:978663. doi: 10.3389/fmolb.2022.978663 (PMC9470956; doi:10.3389/fmolb.2022.978663)
Supplement: Supplementary file 1 [file DataSheet1.DOCX]

Supplementary Material

# Supplementary Tables

Supplementary Table 1. A list of key information for each of the 18 exported PfJDPs. The list provides the previous and new PlasmoDB accession numbers, common names (if known), JDP types, Protein Data Bank (PDB) templates used by SWISS-MODEL (Waterhouse et al. 2018) to predict their structures, and their percentage sequence identity to the template.

| **Previous and New PlasmoDB ID** | **Common Name** | **Type** | **Template** | **Sequence**  **Identity (%)** |
| --- | --- | --- | --- | --- |
| PFA0660w/PF3D7_0113700 | - | II | 6RZY | 100.00 |
| PFB0090c/PF3D7_0201800 | KAHsp40 | II | 6RZY | 61.19 |
| PFE0055c/PF3D7_0501100 | - | II | 6RZY | 67.16 |
| PF10_0378/PF3D7_1038800 | PfJ3 | III | 2CTR | 52.31 |
| PF11_0513/PF3D7_1149600 | - | III | 4J7Z | 53.85 |
| PFB0920w/PF3D7_0220100 | - | III | 4WB7 | 52.31 |
| PFL0055c/PF3D7_1201100 | - | III | 4WB7 | 41.54 |
| PF11_0509/PF3D7_1149200 | RESA3 | IV | 2CTR | 40.00 |
| PF11_0512/PF3D7_1149500 | RESA2 | IV | 4J7Z | 43.08 |
| PFA0110w/PF3D7_0102200 | RESA | IV | 4J7Z | 43.08 |
| PFB0085c/ PF3D7_0201700 | - | IV | 2DN9 | 42.42 |
| PFB0925w/PF3D7_0220400 | - | IV | 4J7Z | 44.62 |
| PF10_0381/PF3D7_1039100 | - | IV | 4J7Z | 43.08 |
| PF11_0034/PF3D7_1102200 | eCiJP | IV | 4J7Z | 43.08 |
| PF14_0013/PF3D7_1401100 | - | IV | 2DN9 | 31.82 |
| PFA0675w/PF3D7_0114000 | - | IV | 2CTR | 45.31 |
| PFE0040c/PF3D7_0500800 | MESA | IV | 4J7Z | 28.57 |
| PFL2550w/PF3D7_1253000 | PfGECO | IV | 4WB7 | 30.77 |

# Supplementary Figures


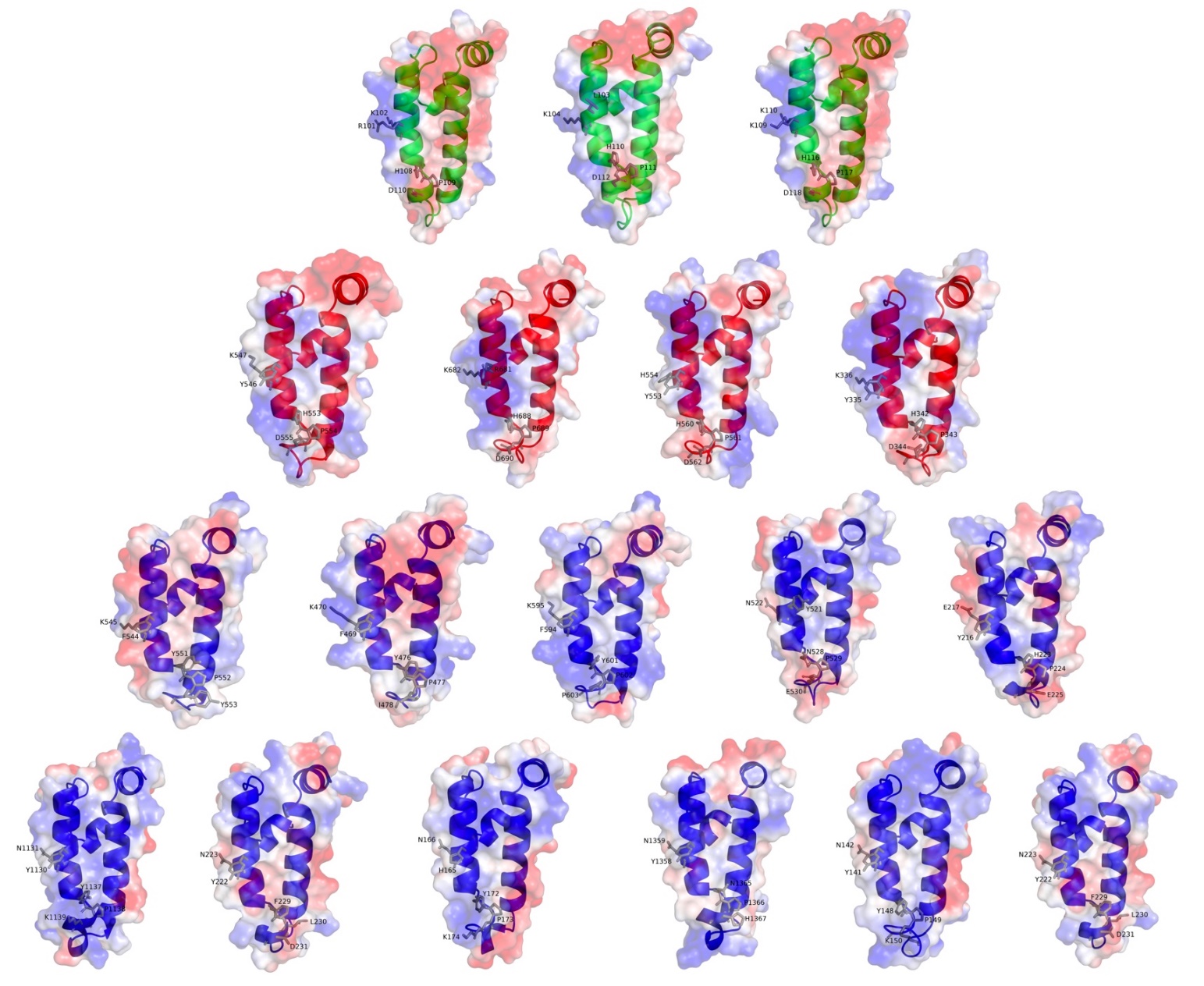


**Supplementary Figure 1.** Three-dimensional models of the J domains of the exported PfJDPs. All of the models are predicted structures, apart from PFA0660w (6RZY; Day et al. 2019). The green, red and blue cartoons refer to the type II, III and IV PfJDPs, respectively. From left to right, the type IIs are PFE0055c, PFA0660w and KAHsp40; the type IIIs are PfJ3, PFB0920w, PFL0055c and PF11_051; and the type IIIs are RESA, RESA2, RESA3, PFB0085c, PFB0925w, PFA0675w, eCiJP, PF14_0013, MESA, PfGECO and PF10_0381. The conserved HPD and RK motifs are shown as grey sticks. The positive charge is shown in blue colored surface, negative charge is shown in red colored surface, and neutral potentials are shown in white colored surface. The surface electrostatic potential was calculated by APBS. The models were prepared using SWISS-MODEL (Waterhouse et al. 2018) and graphically rendered using PyMol 2.5.2 (PyMOL Molecular Graphics System, Version 2.0 Schrödinger, LLC).


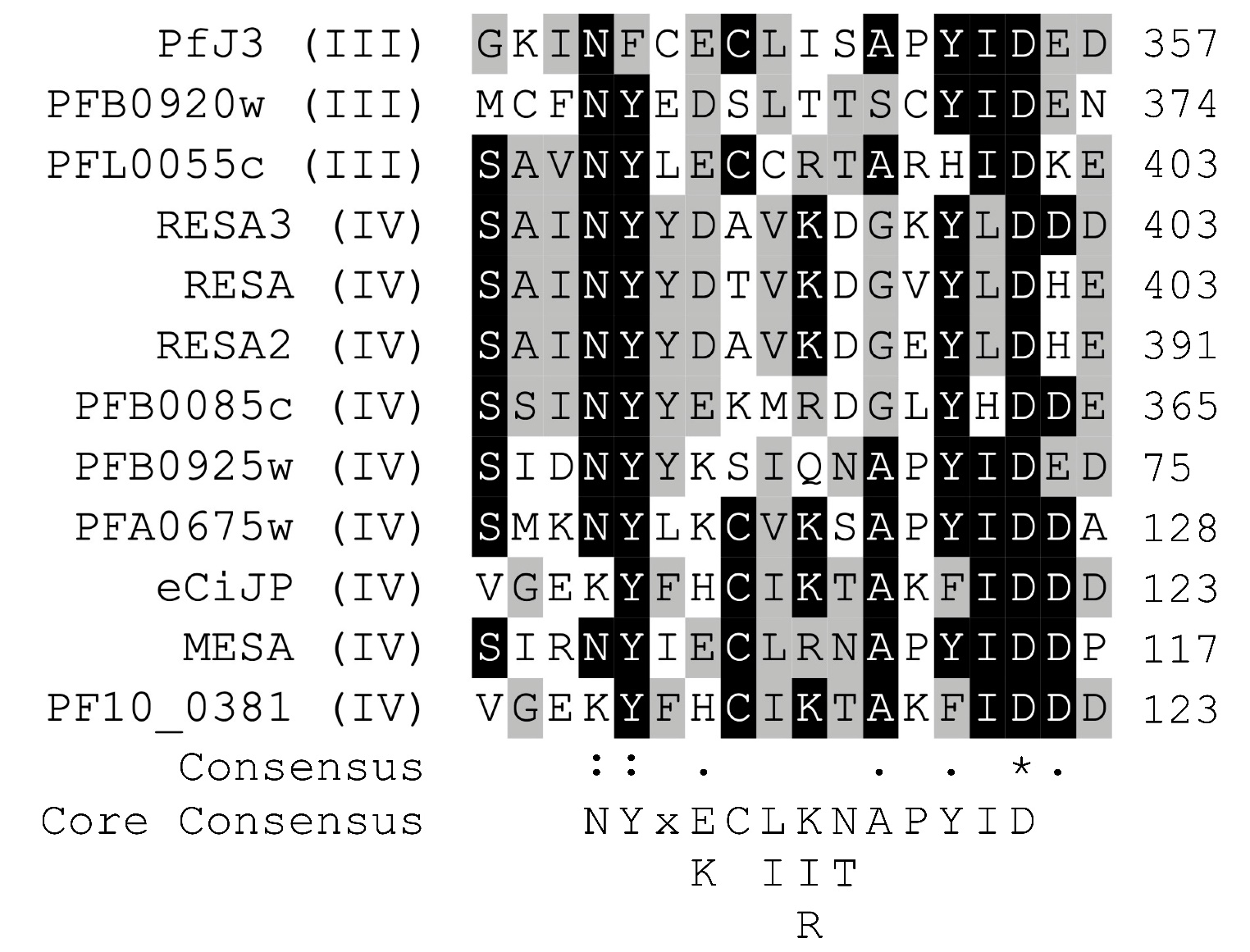


**Supplementary Figure 2.** Multiple sequence alignment of the 12 exported PfJDPs containing the MEC motif. The proteins are defined by either their common name or accession number in the first column, and the roman numerals in brackets refer to the type of JDP. Colored in black are identical amino acids (in at least 50% of the aligned sequences), colored in light grey are similar amino acids (in at least 50% of the aligned sequences), and colored in white are the amino acids with no identity or similarity. The default categories for similar amino acids were applied to the multiple sequence alignment (ILV, FWY, KRH, DE, GAS, P, C and TNQM). The row titled “Consensus” are the common consensus symbols of the multiple sequence alignment: an * (asterisk) indicates positions which have a single, fully conserved residue; a : (colon) indicates conservation between groups of strongly similar properties; and a . (period) indicates conservation between groups of weakly similar properties. The row titled “Core Consensus” refers to 13 residues previously found to be the core of the consensus sequence in MEC motif with the residues defined at the bottom of the alignment in the following order (NYx[E/K]C[L/I][K/I/R][N/T]APYID, where x is any amino acid) including an aspartic acid residue that is shown to be the only amino acid that was highly conserved (Kilili and LaCount, 2011). The alignment was created using Clustal Omega (Sievers and Higgins, 2018) and rendered with box shading using Multiple Align Show (Stothard, 2000).

# References

Day J, Passecker A, Beck HP, Vakonakis I (2019) The Plasmodium falciparum Hsp70-x chaperone assists the heat stress response of the malaria parasite. FASEB J 33:14611–14624

Kilili GK, LaCount DJ (2011) An erythrocyte cytoskeleton-binding motif in exported Plasmodium falciparum proteins. Eukaryot Cell 10:1439–1447

Sievers F, Higgins DG (2018) Clustal Omega for making accurate alignments of many protein sequences. Protein Sci 27:135-145

Stothard P (2000) The sequence manipulation suite: JavaScript programs for analyzing and formatting protein and DNA sequences. BioTechniques 28:1102-1104.

Waterhouse A, Bertoni M, Bienert S, Studer G, Tauriello G, Gumienny R, Heer FT, de Beer TAP, Rempfer C, Bordoli L, Lepore R, Schwede T (2018) SWISS-MODEL: homology modelling of protein structures and complexes. Nucleic Acids Res 46:W296-W303
